# Juice-Based Supplementation Strategies for Athletic Performance and Recovery: A Systematic Review

**DOI:** 10.3390/sports13080269

**Published:** 2025-08-14

**Authors:** Biljana Vitošević, Milica Filipović, Ljiljana Popović, Katarzyna Sterkowicz-Przybycień, Tijana Purenović-Ivanović

**Affiliations:** 1 Faculty of Sport and Physical Education in Leposavić, University of Priština in Kosovska Mitrovica, Dositeja Obradovića bb, 38218 Leposavić, Serbia; biljana.vitosevic@pr.ac.rs; 2 Faculty of Medicine, University of Priština in Kosovska Mitrovica, Anri Didana bb, 38220 Kosovska Mitrovica, Serbia; ljiljana.popovic@med.pr.ac.rs; 3 Department of Gymnastics and Dance, Institute of Sport Sciences, University of Physical Culture in Krakow, 31-571 Krakow, Poland; katarzyna.sterkowicz@awf.krakow.pl; 4 Faculty of Sport and Physical Education, University of Niš, 18101 Niš, Serbia

**Keywords:** juice, athletes, nutrition, exercise, ergogenic aid

## Abstract

The application of natural juices in sports nutrition is attracting growing interest due to their potential antioxidant, anti-inflammatory, and ergogenic properties. Exercise, especially when prolonged or intense, increases oxidative stress and muscle damage, leading athletes to explore dietary strategies that support recovery and enhance performance. This systematic review investigates the effectiveness of five widely studied juices—beetroot, pomegranate, cherry, watermelon, and pickle juice—in the context of athletic supplementation and recovery. A thorough search of the PubMed, Scopus, and Web of Science databases was conducted to identify studies published between 2010 and 2025. Fifty peer-reviewed articles met the inclusion criteria, examining various physiological, biochemical, and performance-related outcomes linked to juice consumption. Given the methodological diversity among studies, a qualitative synthesis was employed. The juices were compared across four key outcomes—inflammation, oxidative stress, delayed onset of muscle soreness, and exercise performance—to determine their most consistent benefits. Beetroot juice, noted for its high nitrate content, consistently enhanced oxygen efficiency and submaximal endurance, although benefits in elite or sprint athletes were less evident. Both pomegranate and cherry juices were effective in reducing muscle soreness and inflammatory markers, particularly when consumed over several days surrounding exercise. Watermelon juice, primarily through its L-citrulline content, offered antioxidant and recovery support, although performance outcomes varied. Evidence for pickle juice was limited, with no notable ergogenic effects beyond anecdotal cramp relief. Overall, natural juices can support recovery and occasionally improve performance, depending on the specific juice, dosage, and athlete characteristics. Beetroot juice stands out as the most reliable in enhancing performance, while pomegranate and cherry juices are more beneficial for recovery. Future research with standardized protocols is essential to determine optimal application across diverse athletic contexts.

## 1. Introduction

The intersection of nutrition and athletic performance is drawing increasing attention, with a growing emphasis on food-based interventions that support recovery, reduce inflammation, and combat oxidative stress [1]. Among these, natural juices have emerged as a promising and accessible strategy, owing to their dense composition of bioactive compounds, such as polyphenols, flavonoids, betalains, vitamins, and amino acids [2]. These constituents are known to influence physiological mechanisms, including antioxidant activity, nitric oxide (NO) bioavailability, and inflammatory pathways—processes that are central to mitigating muscle damage, fatigue, and delayed recovery following intense or prolonged exercise [3,4,5,6].

Despite the proliferation of research into isolated nutrients, there remains significant interest in whole-food sources that offer synergistic effects through multiple compounds [7]. Fruit and vegetable juices—particularly beetroot, pomegranate, cherry, watermelon, and even unconventional options like pickle juice—have been studied in various athletic contexts. While differing in composition, these juices share common proposed mechanisms: the enhancement of endothelial function through NO pathways, the reduction of oxidative stress via radical scavenging, and the modulation of inflammation through cytokine regulation [8,9,10,11,12,13,14,15,16,17,18,19,20]. However, gaps persist in understanding their comparative effectiveness, the optimal dosing strategies, and the consistency of outcomes across populations and exercise modalities. Although they have been researched for some time, we are specifically interested in their current scientific status, including what has been confirmed through robust evidence and where uncertainty remains.

This systematic review seeks to synthesize existing evidence on five commonly studied juices—beetroot, pomegranate, tart cherry, watermelon, and pickle juice—to evaluate their roles in athletic recovery and performance. We aim to explore the extent to which these natural juices improve exercise recovery outcomes through shared or distinct physiological mechanisms. Specifically, we examine whether their bioactive profiles contribute meaningfully to reductions in muscle soreness, inflammation, or oxidative stress and whether this evidence supports their practical use in sports nutrition. Beetroot juice (BRJ), for instance, is rich in dietary nitrates that enhance NO-mediated vasodilation, mitochondrial efficiency, and oxygen delivery to muscles [13,16,19]. Pomegranate juice (POMj) offers potent polyphenols such as ellagitannins, which reduce oxidative stress and modulate cytokines like interleukin-6 (IL-6) and tumor necrosis factor (TNF) [11,14,20]. Tart cherry juice (TCJ), particularly the Montmorency variety, is concentrated in anthocyanins and melatonin, compounds that may reduce muscle damage and promote recovery and sleep [8,9,10,12,15,17,18]. Pickle juice (PJ) has gained attention for its possible neuromuscular effects through transient receptor potential channel activation, which may help relieve exercise-induced muscle cramps via the reflex inhibition of alpha motor neurons [21,22,23]. Finally, watermelon juice (WJ), as a natural source of L-citrulline, supports NO synthesis and improves vascular function while also providing lycopene, beta-carotene, and vitamins A and C [24,25,26,27,28,29,30,31,32,33]. By uniting these interventions under a single analytical framework, this review aims to clarify their physiological underpinnings, assess the strength and limitations of current evidence, and identify where further investigation is warranted. In doing so, we aim to offer both researchers and practitioners a clearer understanding of how these juices may serve as effective tools in enhancing recovery and performance in athletic settings.

## 2. Materials and Methods

### 2.1. Search Strategy

A comprehensive literature search was conducted in early May 2025 across three major databases: PubMed, Scopus, and Web of Science. The search strategy utilized a combination of keywords and Boolean operators (AND/OR), incorporating terms such as (Supplements OR Juices) AND (Athletes OR Recovery OR Exercise Performance), (Beetroot OR Pomegranate OR Cherry OR Pickle OR Watermelon), and (Antioxidants OR Inflammation OR Muscle Soreness OR Cramp).

The search covered the publication period from 2010 to 2025, with the aim of identifying studies that investigated the effects of beetroot, pomegranate, cherry, pickle, and watermelon juices on athletic recovery, exercise performance, muscle soreness, and inflammation.

### 2.2. Inclusion and Exclusion Criteria

The inclusion and exclusion criteria were structured in accordance with the PICOS (Population, Intervention, Comparison, Outcome, Study Design) framework and are presented in Table 1, with the exception of “C” (Comparison), which was not explicitly required, as many nutrition/exercise studies compare outcomes to a placebo or baseline, and our review allowed for heterogeneous comparators [34].

### 2.3. Data Extraction

The study selection process was documented using the Preferred Reporting Items for Systematic Reviews and Meta-analysis (PRISMA) flowchart, which outlined the number of records identified, screened, excluded, and ultimately included in the review [35]. Data extraction was carried out using a standardized form that captured key information, including the first author(s); study design; participant characteristics (age, gender, and training status); type of juice, dosage, duration, and frequency of supplementation; measured outcomes (muscle soreness, inflammation, and performance indicators); and main findings.

This systematic review was reported according to the PRISMA (see Supplementary File S1) and registered in the International Platform of Registered Systematic Review and Meta-analysis Protocols (INPLASY) under the registration number INPLASY202560053 (see Supplementary File S2).

The methodological quality of the included studies was evaluated using the Physiotherapy Evidence Database (PEDro) scale, a validated tool for assessing the quality of clinical research [36]. All authors (B.V., M.F., L.P., K.S.-P., and T.P.-I.) independently performed the quality assessments, with any disagreements resolved through discussion and consensus.

Given the substantial heterogeneity across studies in terms of design, participant characteristics, interventions, and outcomes, a meta-analysis was not feasible. Therefore, a qualitative synthesis was conducted to analyze and compare the findings.

## 3. Results

To ensure methodological transparency and reproducibility, the study identification and selection process was conducted in accordance with the PRISMA guidelines and included two distinct screening phases. A total of 84 records were initially identified through systematic searches of three major databases: PubMed (*n* = 43), Scopus (*n* = 33), and Web of Science (*n* = 8). After removing 12 duplicates, 72 unique records remained and were screened based on their titles and abstracts for relevance to the research topic. At this stage, 10 records were excluded: four involved non-exercising populations, four examined unrelated outcomes, and two focused on elderly or clinical groups rather than healthy, physically active individuals. The remaining 62 articles underwent full-text assessment.

**Figure 1:**
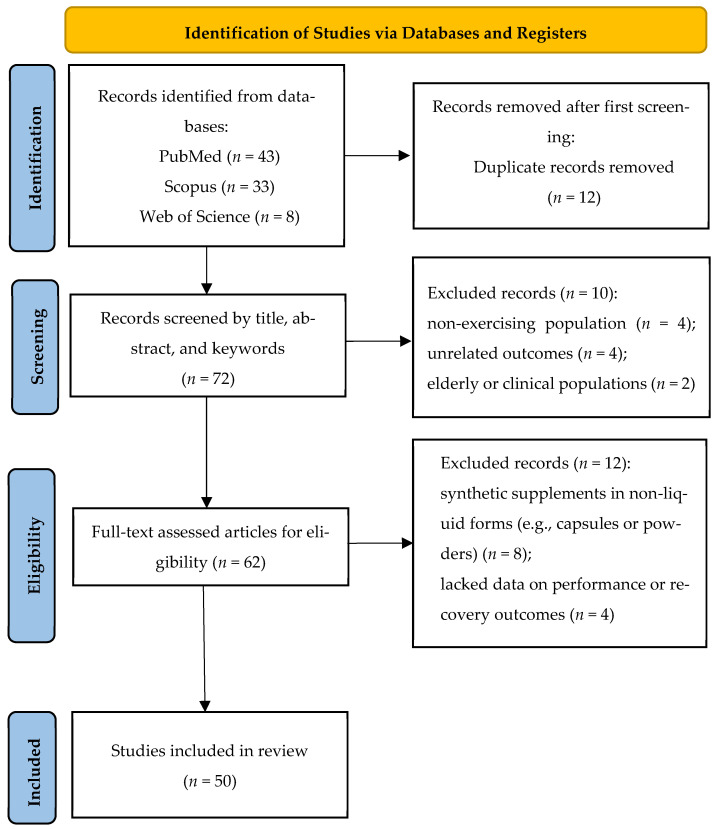
PRISMA flow diagram of the study selection process.

During the full-text screening, 12 additional articles were excluded due to not meeting the inclusion criteria. The most common reason was the use of synthetic supplements in non-liquid forms—primarily powders, capsules, or multi-ingredient formulations not specific to the targeted juices (*n* = 8). The other four exclusions were due to irrelevant outcome measures (e.g., lacking data on physical performance, muscle recovery, inflammation, or oxidative stress). Several of these excluded studies also involved elderly or clinical populations using the supplements for disease-related recovery, rather than exercise-induced stress in athletic or physically active individuals. This two-phase exclusion process ensured that the final qualitative synthesis included only studies strictly aligned with the PICOS criteria (*n* = 50). The full process of study selection is presented in Figure 1.

### 3.1. Methodological Quality Assessment

The methodological quality and internal validity of the included RCTs were assessed using the PEDro scale. This tool examines 11 design-related criteria, such as random allocation, concealed allocation, baseline comparability, blinding procedures, and adequacy of follow-up. While the first item pertains to external validity and is not factored into the final score, the maximum attainable score is 10. Across the included studies, the average PEDro score was 7 out of 10, reflecting an overall good level of methodological quality (see Supplementary Table S1). According to the PEDro classification system, scores ranging from 6 to 8 denote good quality, while scores of 9 or 10 represent excellent quality. The results indicate that the majority of the studies reviewed were well designed, with a limited risk of bias, thereby enhancing the reliability of the findings.

### 3.2. Study Characteristics

Out of the 50 selected studies, 29 investigated the effects of BRJ, and six examined POMj, TCJ, or WJ, while only three focused on PJ’s impact on performance and recovery. The majority of the included studies were randomized, placebo-controlled trials, with many employing crossover or counterbalanced designs to minimize interindividual variability and reduce potential bias. Sample sizes ranged from 9 to 80 participants (a total of 788 participants), with an average age of approximately 20 years. Most study populations comprised young, recreationally active individuals, trained adults, and competitive athletes, with a predominance of male participants (M = 594, F = 194). Research was conducted in a diverse array of countries, including the United Kingdom, the United States, Canada, Iran, Tunisia, China, Australia, Spain, Latvia, the Netherlands, Denmark, Italy, and Poland. This wide geographic distribution underscores the global scientific interest in juice-based supplementation and enhances the generalizability of the findings.

A summary of the selected studies examining the effects of various juice types, ranked by relevance and organized for comparative synthesis, is presented in Table 2, Table 3, Table 4, Table 5 and Table 6. Due to the heterogeneity in terms of dosage, outcome measures, and participant characteristics, studies were divided by juice type to reflect the scope of the literature and support a comprehensive narrative analysis.

## 4. Discussion

This systematic review examined the role of various natural juices in sports supplementation and recovery, highlighting key trends and findings across the literature. The primary objective was to assess the scientific evidence supporting the use of natural juices as nutritional interventions among athletic populations. In light of the increasing interest in functional foods and natural alternatives to synthetic supplements, this topic holds both contemporary relevance and practical importance. Juices enriched with antioxidants and bioactive compounds are often promoted for their potential to reduce oxidative stress, support recovery, and enhance physical performance. However, the extent to which these benefits are supported by empirical evidence remains inconsistent. This review aimed to clarify which effects have been substantiated through scientific research, which expected outcomes have not been reliably demonstrated, and how these juices currently stand as evidence-based supplements within the context of athletic performance and recovery.

### 4.1. Beetroot Juice

Beetroot juice emerged as the most frequently studied supplement among the included articles. It is a rich source of dietary nitrate (NO_3_^−^), which is converted in the body into NO—a molecule recognized for its vasodilatory effects and potential to enhance athletic performance. Through multiple physiological mechanisms, NO enhances oxygen utilization during skeletal muscle contraction, facilitates more even oxygen distribution within muscle tissue, and supports cellular oxygen delivery through the endogenous oxidation of L-arginine [87,88]. Additionally, it has been associated with enhanced mitochondrial efficiency, increased glucose availability, immunomodulatory functions, and the stimulation of mitochondrial biogenesis [89]. Due to these wide-ranging physiological effects, BRJ has been extensively studied for its impacts on cardiovascular function. For instance, Zhang et al. [64] observed a significant reduction in both the average heart rate (HR) and rating of perceived exertion (RPE) following the acute consumption of BRJ containing 6.45 mmol of nitrate. Interestingly, higher doses did not yield further improvements. These results suggest enhanced left ventricular contractility, which may lead to an increased stroke volume. However, the study did not report significant changes in blood pressure (BP), in contrast to the findings of Esen et al. [53] and Wylie et al. [45], who noted BP reductions following BRJ supplementation. Such discrepancies may be attributed to variations in the timing and methodology of BP assessment.

With regard to metabolic markers, Zhang et al. [64] reported no significant changes in blood lactate levels at the maximal exercise intensity, contrasting with findings from other studies [38,45,61], which observed elevated lactate concentrations at supramaximal intensities. These findings suggest that the effect of BRJ on lactate production may be influenced by the degree of exercise-induced muscle hypoxia, which in turn modulates the NO metabolic pathway.

In the context of recovery, Daab et al. [50] found that daily supplementation with 2 × 150 mL of BRJ reduced delayed-onset muscle soreness (DOMS) in semi-professional soccer players following simulated match conditions, despite no corresponding changes in biochemical indicators of muscle damage. Similarly, Clifford et al. [42] reported decreased muscle pain and improved dynamic muscle function after sprint-induced muscle damage; however, there were no observed improvements in sprint performance or isometric strength. In a later study, Clifford et al. [90] found that BRJ had no effect on post-exercise recovery, with no improvements in markers of inflammation or muscle damage. Moreover, Clifford et al. [42] and Esen et al. [54] demonstrated that acute BRJ supplementation failed to reduce post-exercise creatine kinase (CK) and high-sensitivity C-reactive protein (hsCRP) concentrations, despite increasing the pain threshold and exerting analgesic effects—likely attributable to its phenolic compounds and betalain content. These findings support the emerging perspective that the recovery-related benefits of BRJ may involve complex interactions among multiple bioactive components, rather than nitrate metabolism alone.

Exercise performance was the most commonly investigated outcome in BRJ supplementation research. Multiple studies [38,40,42,45,48,49,50,52,53,55,57,58,60] reported improvements in maximal oxygen uptake (VO_2_max), time to exhaustion, and power output when BRJ doses containing 6.4–12.8 mmol of NO_3_^−^ were ingested 2–3 h prior to exercise. However, several studies [38,40,55,59,63] found no significant effects on performance, particularly in sprint-based activities [39,42] and swimming disciplines [49,61].

For example, Jonvik et al. [46] explored the influence of training status on the ergogenic response to BRJ supplementation in sprint athletes. The study included recreational, competitive, and Olympic-level elite participants, all of whom exhibited significant increases in plasma nitrate and nitrite concentrations after six days of BRJ intake. Despite this physiological response, no improvements were observed in peak or mean power output across three repeated Wingate tests. However, all groups experienced a consistent reduction of approximately 2.8% in the time taken to reach peak power, indicating an enhanced acceleration capacity. This benefit from BRJ appeared independent of the training level, suggesting that even elite sprint athletes—typically characterized by a high proportion of type II muscle fibers [91]—may experience performance gains in acceleration-specific contexts. Although the impact on overall power output was limited, improved acceleration may offer a competitive advantage in sports where explosive starts are critical, such as sprinting, speed skating, BMX racing, and various field-based sports. The study [46] concluded that BRJ may hold situational value in enhancing acceleration during high-intensity exercise, regardless of an athlete’s performance level. In a rigorous study involving elite professional tennis players, Fernández-Elías et al. [51] explored whether acute BRJ supplementation could enhance movement patterns and performance during competitive match play. Despite the promising nitrate content (6.4 mmol) in the beetroot shot, the results showed no significant improvements in running performance, serve speed, or grip strength compared to the placebo. The study’s use of GPS and accelerometry to measure real-time match dynamics was a methodological strength, offering the precise tracking of movement under competitive conditions. However, the lack of observed benefits may be due to the athletes’ high training status and possible ceiling effects in their physiological adaptations. Additionally, the study [51] highlights that acute nitrate supplementation may not be sufficient in intermittent sports like tennis, where performance is influenced by complex physical and technical demands.

Interestingly, performance improvements were more consistently observed in endurance-based activities over middle distances (e.g., 1–10 km), especially when BRJ was combined with caffeine. For example, a study combining 70 mL of BRJ (administered 120 min before exercise) with caffeine (6 mg/kg, taken 60 min prior) significantly improved 1000 m running performance and accelerated post-exercise recovery, suggesting a synergistic effect between the two supplements [65].

Christensen et al. [37] investigated the effects of six-day BRJ supplementation (0.5 L/day) in highly trained cyclists. Although the nitrate levels increased, no improvements were observed in oxygen uptake (VO_2_) kinetics, endurance capacity, or repeated sprint performance. The authors proposed that highly trained athletes may already possess optimized endogenous NO production due to the training-induced upregulation of NO synthase enzymes, which are known to increase with training [90]. This may also account for the limited ergogenic benefits reported in elite-level sports such as tennis and swimming.

Conversely, some studies demonstrated positive outcomes, including improved muscle oxygenation and reduced oxygen costs during submaximal exercise efforts [43,56]. Patrician and Schagatay [43] investigated the effects of dietary nitrate supplementation, in the form of concentrated BRJ (70 mL), on arterial oxygen saturation (SaO_2_) following dynamic apnea dives in trained male apnea divers. The study demonstrated that BRJ consumption significantly elevated post-dive SaO_2_ values compared to a placebo, indicating a more efficient oxygen-conserving response. These findings point out that dietary nitrate may enhance safety margins and performance in apnea by reducing the oxygen cost. The authors [43] further propose that such supplementation could benefit other sports involving restricted breathing, such as swimming, spearfishing, or synchronized swimming. Additionally, other research highlighted enhancements in cognitive performance under physical stress [48,63], indicating that BRJ’s benefits may extend beyond muscular function. However, gender-related differences have emerged, with less consistent effects observed among female participants [64].

In summary, BRJ appears to be most effective in supporting endurance and submaximal performance, especially when consumed at doses of 6.4–12.8 mmol of nitrate, approximately 2–3 h prior to exercise. Its efficacy, however, is influenced by factors such as the training status, the type of exercise, and individual variability in responsiveness. Limited or inconsistent effects have been reported in elite athletes and in short-duration, high-intensity exercise formats, such as repeated sprints with rest intervals, which may offset any ergogenic advantages of BRJ. While findings related to recovery and cardiovascular markers are promising, the overall evidence remains mixed, underscoring the need for more standardized, controlled research.

### 4.2. Pomegranate Juice

Pomegranate juice is another polyphenol-rich juice with strong antioxidant properties. In a study by Ammar et al. [69], natural POMj supplementation significantly attenuated acute oxidative stress following a weightlifting session. Compared to a placebo, POMj led to a smaller rise in malondialdehyde—a marker of lipid peroxidation—and a greater increase in enzymatic antioxidant activity immediately after exercise. These protective effects are likely due to POMj’s rich polyphenol profile, which contributes to antioxidant defense through mechanisms such as free radical scavenging, the modulation of antioxidant enzymes, metal ion chelation, and the recycling of vitamins C and E. Additionally, the study [69] noted that oxidative stress was typically more pronounced in the morning, yet POMj supplementation appeared to blunt this time-of-day-dependent response. Overall, POMj appears effective in minimizing oxidative damage and accelerating recovery after intensive resistance training. In a follow-up study, Ammar et al. [71] assessed hormonal and homocysteine (Hcy) responses to Olympic-style weightlifting. Compared to a placebo, POMj supplementation resulted in a lowered acute testosterone response and a significant 14% reduction in the plasma Hcy concentration during the recovery period. While both groups exhibited typical post-exercise hormonal fluctuations, only the POMj group showed a sustained decrease in Hcy, with an inverse correlation observed between the Hcy and testosterone concentrations. These findings suggest that POMj may play a role in modulating both oxidative and endocrine responses to strength-based exercise, although additional research is needed to clarify its broader physiological implications.

Furthermore, Urbaniak et al. [70] investigated the long-term effects of POMj supplementation (50 mL/day over two months) on antioxidant capacity and iron metabolism in elite rowers. The POMj group exhibited a significantly greater resting total antioxidant capacity (TAC) compared to the placebo. Post-exercise, both groups experienced a rise in IL-6 and a drop in TAC, but the POMj group consistently maintained higher antioxidant levels. However, POMj supplementation did not influence iron-related biomarkers, including soluble transferrin receptors, iron levels, or hepcidin. These results support the role of POMj in enhancing antioxidant defense in trained individuals, although it appears to have no effect on iron metabolism.

Trombold et al. [66] examined the impacts of an ellagitannin-rich pomegranate extract (POMx) on recovery following eccentric exercise. Participants who consumed POMx experienced benefits in recovery after isometric muscle strength exercise within 2–3 days compared to the placebo group. However, there were no significant differences in inflammatory or muscle damage biomarkers, such as IL-6, CK, or C-reactive protein (CRP), suggesting that POMx may improve functional muscle recovery, although the mechanisms remain unclear. In another study [67], the authors examined whether POMj supplementation could aid recovery after eccentric exercise in 17 resistance-trained men. Participants consumed either POMj or a placebo before performing intense eccentric elbow and knee exercises. The results showed significantly improved recovery of elbow flexor strength and reduced muscle soreness in the POMj group from 2 to 168 h after exercise, while no such benefits were observed for the knee extensors. Although the precise mechanism remains unclear, the high polyphenol content in POMj may have stabilized muscle cell membranes and preserved excitation–contraction coupling by scavenging peroxyl radicals and limiting lipid peroxidation [92]. This membrane-stabilizing effect could explain the early attenuation of muscle weakness observed at the 2 h mark. These findings reveal a targeted benefit of POMj for upper-body muscle recovery in trained individuals following eccentric loading.

Dosing protocols varied across studies, with pomegranate supplementation ranging from 50 to 1000 mg of extract or 250–500 mL of juice, typically administered over a period of 7 to 15 days. Despite the recognized antioxidant potential of anthocyanins and ellagitannins in pomegranate, the evidence for their effectiveness in supporting muscle recovery and enhancing performance remains mixed. While some studies confirm POMj’s role in mitigating reactive oxygen species, lipid peroxidation, and pro-inflammatory cytokines, the magnitude and consistency of these effects appear to be context-dependent. This variability may stem from differences in study protocols, supplementation strategies, and the training statuses of participants, all of which can modulate the physiological response to pomegranate supplementation.

### 4.3. Cherry Juice

Tart cherries (particularly the Montmorency variety) contain significantly higher concentrations of bioactive compounds—notably anthocyanins and total polyphenols—than sweet cherries. Research demonstrates that TCJ possesses 2–3 times greater antioxidant capacity compared to its sweet counterpart, potentially explaining its more robust anti-inflammatory and recovery-enhancing effects in exercise contexts [15,17]. This enhanced antioxidant activity stems primarily from elevated levels of cyanidin-3-glucoside and cyanidin-3-rutinoside, two key anthocyanins that mediate free radical scavenging and inflammatory pathway modulation [92,93]. Connolly et al. [8] were the first to examine its effects on exercise-induced muscle damage, reporting that CJ significantly reduced strength loss and muscle pain, although it had no effect on tenderness or range of motion. These benefits were attributed to CJ’s ability to reduce secondary muscle damage by attenuating the inflammatory response. The juice administered in the study was equivalent to the consumption of 100–120 fresh cherries per day, which likely contributed to its efficacy. The proposed biological mechanism involves protecting against the secondary damage cascade that follows eccentric muscle contractions. Such contractions initially cause the mechanical disruption of myofibrils and cell membranes. If extensive, this damage triggers an inflammatory response involving leukotrienes, neutrophil infiltration, and free radical generation—factors that further exacerbate tissue injury. The bioactive compounds in TCJ may help interrupt this cycle, thereby preserving muscle structure and function.

Kuehl et al. [94] found that daily supplementation with 355 mL of Montmorency CJ for 7 days before and during a marathon significantly reduced post-race muscle soreness. Bell et al. [73] reported improvements in cycling economy and the preservation of muscle strength during a 72 h recovery period in trained cyclists, along with significant reductions in inflammatory markers such as IL-6 and hsCRP when compared to a placebo. Similarly, Bowtell et al. [72] observed the improved recovery of isometric muscle strength and reduced oxidative stress indicators (e.g., CRP and CK) following strenuous exercise. However, findings across the literature are not entirely consistent. Several studies found no significant effects on athletic performance, muscle soreness, or recovery outcomes [75,76,77]. Notably, a study involving professional soccer players [76] failed to demonstrate clear recovery benefits from TCJ supplementation, suggesting that the effects of CJ may vary depending on the sport, training status, or exercise protocol.

The dosages used across the reviewed studies varied considerably, typically ranging from 60 to 300 mL per day, which is approximately equivalent to 100–180 cherries, and were generally administered before and/or after exercise. However, inconsistencies in antioxidant-monitoring methods, differences in exercise intensity, and variations in the supplementation duration and timing of intake complicate cross-study comparisons.

In summary, although TCJ appears to offer potential benefits for recovery, which has already been established [95]—particularly in preserving muscle function and reducing inflammation when consumed for several days pre-exercise—the current body of evidence is not yet strong enough to classify it as consistently effective across all contexts or athletic populations.

### 4.4. Pickle Juice

Pickle juice, albeit an unconventional supplement, has garnered interest mainly for its potential role in muscle cramp prevention and its electrolyte replenishment properties. It is commonly believed that dehydration and sodium loss contribute to the onset of exercise-associated muscle cramps. McKenney et al. [80] examined the effects of consuming one or two boluses of PJ during exercise on plasma electrolytes and hydration status. The study involved nine euhydrated, physically active men exercising in a hot environment and compared the effects of no PJ intake, a single bolus (1 mL/kg), and two boluses. The results showed that the plasma sodium and potassium concentrations remained unaffected, with no signs of hyperkalemia or fluid imbalance. Additionally, plasma osmolality and volume also remained unchanged. These findings support the notion that consuming up to two small servings of PJ during exercise is safe and does not disrupt electrolyte or fluid homeostasis; however, as the study did not assess the muscle cramp incidence, its effectiveness for cramp prevention remains inconclusive.

Similarly, Miller et al. [78] assessed whether the ingestion of PJ, mustard, or deionized water under hypohydrated conditions affected plasma electrolyte levels or osmolality. After inducing approximately 2.9% hypohydration through two hours of vigorous exercise, participants consumed one of the test fluids. Despite the intake of around 1.5 g of sodium from PJ or mustard, no significant changes were observed in plasma sodium or potassium levels, osmolality, or plasma volume within 60 min. These results indicate that neither PJ nor mustard exacerbates exercise-induced hypertonicity or causes hyperkalemia, but they are also ineffective as rapid electrolyte or fluid replenishment strategies.

Peikert et al. [79] investigated the effects of the pre-exercise ingestion of PJ, hypertonic saline, and deionized water on aerobic performance and thermoregulatory responses. The study showed no significant differences among the three beverages in the time to exhaustion, core temperature, sweat volume, or plasma volume. While the core temperature increased during exercise, the rise was consistent across all groups. The authors [79] proposed that larger volumes of PJ might be required to elicit measurable physiological effects. Although concerns exist regarding hyperkalemia—given its association with fatigue and potential cardiac arrhythmias [96]—this study found no causal link between PJ intake and elevated potassium levels. Minor increases in plasma potassium were likely due to exercise-induced muscle contractions rather than the ingested fluids.

Overall, the current evidence does not support ergogenic or thermoregulatory benefits of PJ supplementation in athletic performance or recovery. Although anecdotal reports suggest a potential role in cramp relief, the scientific validation of this claim remains limited. Further research is warranted to clarify PJ’s mechanisms of action and its practical effectiveness, particularly in real-world athletic settings.

### 4.5. Watermelon Juice

Watermelon juice, recognized for its high L-citrulline content, has shown promising but varied effects on exercise performance and recovery. Although its influence on immediate performance enhancement remains uncertain, several studies suggest that regular consumption may contribute to faster recovery, reduced blood lactate concentrations, and the preservation of muscle function post-exercise. Multiple studies demonstrated that chronic supplementation, such as 500 mL over a six-week period, was associated with enhanced total antioxidant capacity, reduced muscle soreness, and improved VO_2_max [86]. Similarly, citrulline-enriched WJ was found to improve muscle oxygenation, maintain jump performance, and lower plasma lactate levels after a half-marathon [84], while Bailey et al. [83] observed improved oxygen delivery to the muscles, although no significant effect was found on time to exhaustion.

Acute or short-term supplementation, however, produced less consistent outcomes. Cutrufello et al. [82] found no improvements in VO_2_max, anaerobic threshold, or time to exhaustion following a single pre-exercise dose. Likewise, in another study [85], seven days of WJ supplementation did not enhance isometric strength, barbell velocity, muscle oxygenation, or vascular diameter during resistance exercise in trained men, although a small increase in the percentage change of muscle oxygenation was noted. The form in which the juice is consumed also appears to affect its efficacy. Pasteurization was found to reduce the bioavailability of citrulline, suggesting that fresh or minimally processed juice may be more effective [81].

In summary, WJ appears to be more effective as a recovery aid and antioxidant booster when consumed regularly and in sufficient quantities. Its effectiveness as a short-term ergogenic supplement in improving strength or endurance performance remains less certain.

### 4.6. Comparative Discussion of Physiological Outcomes

Each juice investigated in this review exhibits distinct strengths and limitations across key physiological domains relevant to athletic recovery and performance, spanning oxidative stress, inflammation, DOMS, and exercise capacity (see Table 7).

Due to its nitrate content, BRJ consistently supports submaximal performance and oxygen efficiency, particularly in endurance exercise. However, its effects on inflammation, DOMS, or recovery markers are inconsistent across studies and tend to diminish in elite populations with preexisting optimized NO pathways [37,45,46,61,64]. POMj shows greater consistency in reducing oxidative stress and inflammation, attributable to its high polyphenol and ellagitannin content. While the performance benefits are less robust, its ability to modulate hormonal and inflammatory responses after strength training is promising [66,68,69,71]. CJ shares a similar anti-inflammatory profile with POMj. Multiple studies report reductions in IL-6, CRP, and DOMS [73,74,75], particularly with pre- and post-exercise supplementation. However, its effects on objective performance metrics (e.g., sprint time or power output) are more variable [72,73,74]. WJ, rich in L-citrulline, can improve antioxidant capacity and reduce post-exercise lactate, although most benefits require chronic use rather than acute intake [83,84,86]. Its impact on actual performance outcomes remains less established. PJ, while historically used for cramp relief, lacks strong evidence in terms of inflammation, DOMS, or recovery benefits. Most studies confirm its safety in moderate doses but demonstrate no significant effects on electrolyte balance, plasma volume, or muscle function [78,79,80].

Overall, POMj and CJ demonstrate the strongest recovery-related benefits (including anti-inflammatory and antioxidant effects), whereas BRJ offers the most consistent ergogenic effects for endurance. WJ supports the antioxidant status with prolonged use, and PJ’s utility remains primarily anecdotal. The variability in the results highlights the importance of tailoring supplementation according to the exercise type, athlete training status, and timing protocols.

### 4.7. Practical Implications for Application

BRJ shows the most consistent performance-enhancing effects, especially for submaximal endurance exercise, with recommended doses between 6.4 and 12.8 mmol of nitrate, typically consumed 2–3 h before activity [38,45,64]. However, its efficacy may diminish in elite athletes, potentially due to ceiling effects in NO-related pathways, as demonstrated by Fernández-Elías et al. [51], who reported no benefit from acute BRJ use in professional tennis players. Moreover, nitrate supplementation may be less effective in intermittent sports (e.g., tennis, soccer), where performance depends not just on aerobic efficiency but also on technical–tactical factors.

To reduce inflammation and oxidative stress, POMj and TCJ are more reliable. POMj is typically consumed at 250–500 mL/day for 7–15 days and is particularly effective after resistance or strength-based training, where it modulates cytokines and oxidative markers [68,69,71]. TCJ, usually dosed at 60–300 mL/day for several days before and after exercise, optimally reduces DOMS and preserves muscle function following eccentric or prolonged efforts [72,73,74].

WJ, due to its L-citrulline content, supports antioxidant defenses and may reduce soreness, but its effects require chronic supplementation (≥1–2 weeks, 500–710 mL/day). Acute benefits on strength or endurance remain unclear [83,84,86]. PJ lacks evidence in terms of ergogenic or recovery benefits; its role in cramp relief remains anecdotal, and it is typically used in small, acute doses (1–2 mL/kg) during or after exercise [78,80].

Target populations differ: BRJ suits trained recreational or sub-elite endurance athletes, while POMj and TCJ benefit athletes in high-volume/-intensity resistance training. Supplementation strategies should align with the exercise type, training status, timing, and goals. Future research should address optimal protocols, gender-specific responses, and potential juice–nutrient synergies.

## 5. Conclusions

This systematic review emphasizes the diverse physiological effects and supplementation outcomes associated with various natural juices in athletic settings. A key shared feature among these juices is their rich content of bioactive compounds such as nitrates, polyphenols, anthocyanins, citrulline, and electrolytes. These components play a role in alleviating oxidative stress, mitigating muscle damage, and supporting post-exercise recovery.

Among the juices examined, BRJ consistently demonstrated ergogenic benefits, particularly for endurance and submaximal exercise, when consumed at optimal doses and timing. However, its effectiveness may diminish in elite athletes due to physiological ceiling effects, and its application appears limited in technically demanding, intermittent sports. POMj and TCJ showed the strongest evidence for anti-inflammatory and antioxidant effects, especially when consumed prophylactically over multiple days before and after exercise. These juices are particularly effective for recovery from resistance training and eccentric exercise. While some performance improvements were noted, they were less reliably observed across studies. Watermelon juice appeared more effective in enhancing recovery and boosting the antioxidant status when consumed over extended periods, rather than as an acute performance enhancer. Conversely, pickle juice did not show measurable benefits for performance, hydration, or electrolyte replacement, and current support for its role in preventing muscle cramps remains largely anecdotal.

The variability in study designs, participant demographics, supplementation protocols, and outcome measures highlights the need for more standardized and rigorous research to determine optimal supplementation strategies and their sport-specific applications. Given their rich profiles of bioactive compounds, natural fruit and vegetable juices offer promising benefits as dietary supplements for athletic performance and recovery. However, their application should be tailored based on the type, intensity, duration, and goals of the physical activity. Each juice contains a unique composition of phytonutrients that exert effects through specific metabolic pathways and mechanisms of action. Therefore, future research should focus on identifying the precise bioactive constituents responsible for the observed physiological outcomes, as well as understanding their pharmacokinetics and interactions. Moreover, the optimal dosing regimens and threshold levels required to elicit beneficial effects remain to be clearly defined. The potential synergistic or antagonistic effects of combining various juices or compounds should also be explored to better inform evidence-based supplementation strategies for athletes and physically active individuals. Finally, when tailored to individual needs, juice supplementation may offer a natural and functional strategy to enhance recovery and support athletic performance.

### Limitations and Future Perspectives

Although interest in natural juice supplementation to enhance athletic performance and recovery continues to grow, this systematic review has several notable limitations. One major challenge was the heterogeneity among the included studies. Differences in dosage, duration of supplementation, juice formulation (e.g., concentrates vs. fresh juices), and the types of outcome measures used made it difficult to directly compare findings across studies. This variability also prevented the performance of a meta-analysis. Another limitation was the relatively small sample sizes in many of the studies, along with a lack of representation of female athletes and diverse athletic populations. This restricts the generalizability of the findings across sexes and training backgrounds. Moreover, the range of exercise modalities studied, including endurance, resistance, and sprint-based protocols, along with inconsistencies in supplementation timing (acute vs. chronic use), further challenged the ability to draw firm conclusions about the efficacy of each juice type. The absence of standardized approaches for the evaluation of recovery, oxidative stress, and performance outcomes also hindered the synthesis of results across trials.

To advance the field, future research should prioritize several key areas:

- Conducting well-powered RCTs with larger sample sizes and standardized supplementation protocols;
- Designing comparative studies that directly assess different juice types using consistent methodologies;
- Implementing long-term intervention studies to examine chronic adaptations and recovery processes;
- Exploring the underlying cellular and molecular mechanisms, especially for lesser-understood juices like pomegranate and pickle juice;
- Including female participants and athletes from a variety of backgrounds to assess sex- and population-specific responses;
- Combination strategies (e.g., juice plus caffeine or other nutrients) to evaluate synergistic effects on performance and recovery.

Clarifying dose–response relationships, identifying optimal timing strategies, and understanding formulation choices (e.g., fresh vs. pasteurized, juice vs. extract) will also be essential in developing evidence-based guidelines in sports nutrition.

## Figures and Tables

**Table 1:** Inclusion and exclusion criteria used during full-text screening.

Category	Criteria	Details
Inclusion	Population	Human participants who are athletes or physically active individuals.
	Intervention	Supplementation with beetroot, pomegranate, cherry, watermelon, or pickle juice in liquid form.
	Study Design	Randomized controlled trials (RCTs), cohort studies, cross-sectional studies, case studies.
	Outcomes	Studies reporting at least one outcome related to exercise performance, muscle soreness, inflammation, or recovery.
	Language	Published in English.
Exclusion	Population	Animal studies.
	Publication type	Meta-analyses, systematic reviews, narrative reviews, or other secondary research.
	Supplement type	Studies using synthetic or encapsulated supplements (e.g., pills, powders) or multi-ingredient formulations unrelated to the juice itself.
	Outcomes	Studies that did not report performance or recovery-related outcomes.

**Table 2:** Comprehensive overview of studies about beetroot juice-based supplementation (*n* = 29).

Study	Study Design	Participants’ Characteristics	Juice Type	Dosage	Measured Outcomes	Findings
Christensen et al. (2012) [37]	Randomized, crossover	10M elite endurance cyclists	BRJ (nitrate-rich)	6 days, 500 mL/day, 0.5 g of NO_3_^−^	VO_2_ kinetics, endurance TT, repeated sprint capacity	Plasma nitrate levels were significantly elevated in BRJ conditions. No significant improvements in VO_2_ kinetics, TT performance, or repeated sprint capacity. The findings suggest that nitrate supplementation may not confer ergogenic effects in highly trained athletes.
Wylie et al. (2013) [38]	Randomized, double-blind, crossover	14M recreational team-sport players	BRJ (nitrate-rich)	490 mL, 4.1 mmol of NO_3_^−^ per 70 mL, ~30 h before exercise	YYIR1, blood lactate, plasma nitrite concentration, muscle glucose uptake, muscle excitability	BRJ supplementation resulted in a 4.2% improvement in YYIR1 performance. Blood lactate was not significantly different between groups. Mean blood glucose was lower and plasma nitrite was higher in the BRJ group compared to placebo.
Martin et al. (2014) [39]	Randomized, double-blind, crossover	16 team-sport athletes (9M + 7F)	BRJ (nitrate-rich)	70 mL, 0.3 g of NO_3_^−^, SD, 2 h before repeated sprint protocol	Sprint performance, total work done, power output	BRJ supplementation did not improve repeated sprint performance, total work done, or power output. The study suggests that nitrate supplementation may not be beneficial for near-maximal and frequent sprint efforts.
Pinna et al. (2014) [40]	Randomized, crossover design	14M moderately trained master swimmers	BRJ	6 days, 0.5 L/day, 5.5 mmol of NO_3_^−^	Workload at anaerobic threshold, AEC, VO_2_, VCO_2_, VE	The workload at the anaerobic threshold was significantly increased after BRJ supplementation. The aerobic energy cost was significantly reduced in the BRJ supplementation test. Other variables did not show statistically significant differences between the BRJ and control tests, suggesting that it positively affects swimming performance by reducing AEC and increasing the anaerobic workload.
Thompson et al. (2015) [41]	Randomized, double-blind, crossover	16M team-sport players	BRJ	7 days, 140 mL/day, 6.4 mmol of NO_3_^−^	Intermittent sprint performance, cognitive reaction time, total work done	BRJ improved work done during sprints and maintained cognitive reaction time.
Clifford et al. (2016) [42]	Independent groups, randomized	20M team-sport players	BRJ	250 mL, twice a day, 3 days post-exercise	MIVC, CMJ, RI, PPT, CK, hsCRP, PC, LOOH	BRJ reduced decrements in CMJ and RI but had no effect on sprint performance or oxidative stress markers.
Patrician and Schagatay (2016) [43]	Randomized, crossover	14M healthy apnea divers	BRJ (nitrate-rich)	70 mL, ~5.0 mmol of NO_3_^−^, SD, 2.5 h before dynamic apnea performance	Arterial SaO_2_, HR	BRJ supplementation elevated SaO_2_ after 75 m dynamic apnea dives, indicating a potential oxygen-conserving effect. No significant effect on HR.
Thompson et al. (2016) [44]	Randomized, double-blind, crossover	36M team-sport players	BRJ (nitrate-rich)	5 days, 70 mL/day, 6.4 mmol of NO_3_^−^	Sprint performance (5 m, 10 m, 20 m), YYIR1 performance, cognitive reaction time	BRJ supplementation improved sprint split times and distance covered in the YYIR1 test. Reaction time to cognitive tasks was also shorter after BRJ compared to placebo.
Wylie et al. (2016) [45]	Randomized, placebo-controlled, crossover	10M team-sport players	BRJ (nitrate-rich)	5 days, 1 or 2 × 70 mL, 8.2 mmol NO_3_^−^ per day (~4.1 mmol of NO_3_^−^ per 70 mL)	Power output during 24 × 6 s sprints, 7 × 30 s sprints, and 6 × 60 s efforts; blood lactate	BRJ improved power output during short, high-intensity sprints but not during longer intervals or with longer recovery periods. Blood lactate levels increased significantly in the short-duration sprint protocol.
Jonvik et al. (2018) [46]	Randomized, double-blind, crossover	52M: 20 recreational cyclists, 22 national talent speed skaters, 10 Olympic-level track cyclists	BRJ (nitrate-rich)	140 mL/day, ~800 mg/d of NO_3_^−^	DEXA, Wingate test, plasma nitrate and nitrite concentrations, RPE, HR	BRJ significantly increased plasma nitrate and nitrite concentrations in all groups, regardless of training status. No improvement in peak or mean power output across three 30 s Wingate sprints. The only performance benefit observed was a 2.8% reduction in time to reach peak power, suggesting improved acceleration across all athletic levels and sprints.
Richard et al. (2018) [47]	Double-blind, placebo-controlled, crossover	9 international-level short-track speed skaters (4M + 5F)	BRJ	HI: 115 mL, ~6.5 mmol of NO_3_^−^ or LO: 115 mL, ~0.9 mmol of NO_3_^−^, 5 days with DD on day 5	1000 m TT performance; plasma nitrate and nitrite, lactate concentrations	Nitrate supplementation increased plasma nitrate and nitrite but did not improve single or repeated 1000 m TT performance in elite speed skaters. No effect on lactate concentration or recovery.
Thompson et al. (2018) [48]	Randomized, crossover, placebo-controlled	30 recreationally active subjects (18M + 12F)	BRJ	70 mL (~6.4 mmol of NO_3_^−^ per 70 mL) in the morning and in the evening	VO_2_ peak, time to task failure, lactate, phosphocreatine recovery	BRJ enhanced exercise capacity adaptations more than KNO_3_ and SIT alone.
Esen et al. (2019) [49]	Randomized, double-blind, crossover	10 moderately trained swimmers (5M + 5F)	BRJ (nitrate-rich)	3 days, 140 mL/day, ~8 mmol/day of NO_3_^−^	SBP, blood lactate concentration, plasma nitrate and nitrite concentrations, 200 m and 100 m swimming TT	BRJ supplementation increased plasma nitrite concentration and lowered SBP, but had no effect on swim performance. BRJ does not show an ergogenic effect in moderately trained swimmers.
Daab et al. (2020) [50]	Randomized, double-blind, crossover	13M soccer players	BRJ	150 mL, 250 mg of NO_3_^−^, total phenolic content: 385 mg/GAE/L, twice a day for 7 days	SJ, CMJ, MVC, 20 m sprint, CK, LDH, CRP, DOMS	BRJ supplementation reduced perceived muscle soreness and maintained better performance during recovery in CMJ, MVC, and sprint/placebo. No significant effect on biochemical markers of muscle damage.
Fernández-Elías et al. (2020) [51]	Randomized, double-blind	9M professional tennis players	BRJ	70 mL, 6.4 mmol of NO_3_^−^, 3 h before match	Match-play running performance, serve speed, isometric handgrip strength	No significant differences were found between the BRJ and placebo trials in match-play running performance, serve speed, or isometric handgrip strength. Acute BRJ supplementation did not produce any performance benefit in professional tennis players.
Garnacho-Castaño et al. (2020) [52]	Randomized, double-blind, crossover	12M well-trained CrossFit practitioners	BRJ (nitrate-rich)	140 mL, ~12.8 mmol of NO_3_^−^, SD	Number of repetitions, cortisol response, SaO_2_, muscular fatigue	BRJ increased the number of repetitions only in the exercise routine with a 3 min rest between sets. It also caused a greater increase in cortisol and a significant drop in SaO_2_, indicating increased muscular fatigue.
Esen et al. (2022) [53]	Randomized, double-blind, crossover	12M recreationally active	BRJ (nitrate-rich)	140 mL, 12.8 mmol of NO_3_^−^, SD	YYIR1 performance, VO_2_ at sub-maximal and peak levels	BRJ supplementation improved YYIR1 performance but did not significantly affect VO_2_ at sub-maximal or peak levels.
Esen et al. (2022) [54]	Randomized, double-blind, crossover	16 healthy active young adults (10M + 6F)	BRJ (nitrate-rich)	5 days, 2 × 70 mL/day,~12.8 mmol NO_3_^−^/day)	Plasma NO_2_^−^, MVC, RPE	Nitrate-rich BRJ increased plasma NO_2_^−^ concentration and improved RPE in comparison to placebo group, while there was no significant difference in the MVC. BRJ may have implications in enhancing skeletal muscle contractile function.
Giv et al. (2022) [55]	Experimental, control group in pre- and post-test	40M soccer players	BRJ	8 weeks, 100 mL (300 mg of NO_3_^−^)	Aerobic power, respiratory exchange ratio, anaerobic threshold, anaerobic power, field performance, fatigue index	Soccer training combined with BRJ supplementation significantly improved aerobic power, respiratory exchange ratio, anaerobic threshold, anaerobic power, and field performance.
Huang et al. (2022) [56]	Randomized, double-blind, crossover	80 young active winter triathletes (44M + 36F)	BRJ	7 days, 3 doses per day, 6.5 mmol of NO_3_^−^ per 70 mL	VO_2_, respiratory exchange ratio, blood lactic acid, TTE, 10 km XC skiing performance	BRJ supplementation decreased VO_2_, respiratory exchange ratio, and blood lactic acid levels during high-speed running. Increased TTE during cycling exhaustion testing but did not improve 10 km XC skiing performance. BRJ may enhance running economy and cycling TTE but not XC skiing performance.
Jurado-Castro et al. (2022) [57]	Randomized, double-blind, crossover	14F physically active	BRJ	70 mL, 400 mg of NO_3_^−^	CMJ, back squat test, muscular endurance test	Greater CMJ, increased performance. Confirmed ergogenic effect of BRJ on muscular endurance in the lower limbs.
Tan et al. (2022) [58]	Randomized, double-blind, crossover	14M recreationally active	BRJ	4 days, 2 × 70 mL, ~5.9 mmol of NO_3_^−^ per 70 mL	Bench press, back squat, RTF, TSI	Acute BRJ ingestion increased RTF in bench press but not in back squat. No significant change in muscle oxygenation.
Esen et al. (2023) [59]	Randomized, counterbalanced, double-blind, placebo-controlled, crossover	12M trained rugby players	BRJ (nitrate-rich)	140 mL, 12.8 mmol of NO_3_^−^, SD	Modified YYIR1, CMJ, plasma NO_3_^−^ and NO_2_^−^ concentrations	Acute BRJ supplementation increased plasma NO_3_^−^ and NO_2_^−^ but did not improve intermittent running performance or CMJ performance in trained rugby players.
Hemmatinafar et al. (2023) [60]	Randomized, double-blind, crossover	12F young volleyball players	BRJ	50 mL, 4.1 mmol of NO_3_^−^, 8 servings over 2 days	Wall-sit performance, V sit and reach, vertical jump height, pressure pain threshold, thigh swelling, perceived muscle soreness, VAS	BRJ supplementation improved wall-sit performance, reduced swelling, and perceived muscle soreness but did not significantly affect vertical jump height or V sit and reach performance.
Moreno-Heredero et al. (2024) [61]	Randomized, placebo-controlled, double-blind, crossover	18 competitive swimmers (9M + 9F)	BRJ	70 mL, 6.4 mmol/400 mg of NO_3_^−^, SD	100 m swim time, lactate, RPE, TQR	No significant improvement in swimming performance, lactate, or subjective measures. Possible positive effect on exercise.
Neteca et al. (2024) [62]	RCT study	18F endurance athletes	BRJ (nitrate-rich)	50 mL, ~6.2 mmol of NO_3_^−^	VO_2_max, HR, VE, ventilation to oxygen consumption ratio (VE/VO_2_) (VE/VCO_2_)	Significant improvements in VE, respiratory equivalents, and HR that confirm the effective ergogenic potential of BRJ.
Tan et al. (2024) [63]	Randomized, double-blind, crossover	15F team-sport athletes	BRJ (nitrate-rich)	140 mL, 12 mmol of NO_3_^−^, SD	10 m and 20 m sprints, isokinetic handgrip dynamometry, MBT, horizontal CMJ, YYIR1, oral microbiota composition, cognitive flexibility	Acute BRJ ingestion increased plasma NO_3_^−^ and NO_2_^−^ but did not impact exercise performance, cognitive flexibility, or oral microbiota composition.
Zhang et al. (2024) [64]	Randomized, double-blinded, placebo-controlled, crossover	13F recreationally active	BRJ (nitrate-rich)	SD: 6.45 mmol of NO_3_^−^, DD: 12.9 mmol of NO_3_^−^	HR, BP, blood lactate, oxygen saturation, RPE	Acute nitrate ingestion reduced HR and RPE during high-intensity interval exercise but no additional benefit observed with higher nitrate content.
Liu et al. (2025) [65]	Randomized, crossover	20M participants	BRJ	70 mL, 6.4 mmol of NO_3_^−^	Left-hand grip strength, average VJH, HR, blood lactate, BF%	BRJ supplementation along with caffeine may improve 1000 m performance.

Legend: M—male, F—female, BRJ—beetroot juice, NO_2_^−^—nitrite, NO_3_^−^—nitrate, VO_2_—oxygen uptake, TT—time trial, YYIR1—Yo-Yo intermittent recovery level 1, SD—single-dose, DD—double-dose, AEC—aerobic energy cost, VCO_2_—carbon dioxide production, VE—minute ventilation (pulmonary ventilation), MVC—maximal voluntary contraction, MIVC—maximal isometric voluntary contractions, SJ—squat jump, CMJ—countermovement jump, RI—reactive strength index, PPT—pressure pain threshold, CK—creatine kinase, CRP—C-reactive protein, hsCRP—high-sensitivity C-reactive protein, PC—protein carbonyls, LOOH—lipid hydroperoxides, SaO_2_—oxygen saturation, HR—heart rate, DEXA—dual X-ray absorptiometry, RPE—rating of perceived exertion, HI—high, LO—low, KNO_3_—potassium nitrate, SIT—sprint interval training, BP—blood pressure, SBP—systolic blood pressure, GAE—gallic acid equivalent, LDH—lactate dehydrogenase, DOMS—delayed onset of muscle soreness, TTE—time to exhaustion, XC—cross-country, RTF—repetitions to failure, TSI—tissue saturation index, VAS—visual analog scale, TQR—total quality recovery, VO_2_max—maximal oxygen uptake, MBT—medicine ball throw, VJH—vertical jump height, BF%—body fat percentage.

**Table 3:** Comprehensive overview of studies about pomegranate juice-based supplementation (*n* = 6).

Study	Study Design	Participants’ Characteristics	Juice Type	Dosage	Measured Outcomes	Findings
Trombold et al. (2010) [66]	Randomized, crossover	16M recreationally active	POMj (POMx, ellagitannin-rich)	9 days, 500 mL twice a day, 650 mg of polyphenols: 95.5% ellagitannins, 3.5% ellagic acid, and 1% anthocyanins	Isometric strength, muscle soreness, serum markers (CK, myoglobin, IL-6, CRP)	POMx significantly improved recovery of isometric strength at 48 and 72 h post-exercise compared to placebo. Serum markers of inflammation and muscle damage did not differ significantly between conditions.
Trombold et al. (2011) [67]	Randomized, crossover	17M resistance-trained	POMj	250 mL before eccentric exercise, 1.979 mg/L of tannins, 384 mg/L of anthocyanins, and 121 mg/L of ellagic acid derivatives	Isometric strength, muscle soreness (elbow flexors, knee extensors)	POMj reduced soreness and maintained strength in elbow flexors but had no effect on knee extensors.
Ammar et al. (2016) [68]	Crossover design with placebo, clinical trials	9M elite weightlifters	POMj	3 × 1500 mL per day in 48 h, 2.56 g of polyphenols in each 500 mL	Weightlifting performance, RPE, DOMS, HR, SBP, CK, LDH, ASAT, CRP	POMj supplementation reduced muscle soreness, inflammation, and muscle damage responses, while also enhancing performance and accelerating recovery of several biological markers. However, CK, LDH, and ASAT remained elevated, indicating incomplete recovery.
Ammar et al. (2017) [69]	Randomized, crossover	9M elite weightlifters	POMj	500 mL, 2.56 g of TPC, 1.08 g of o-DPO, 292.6 mg of flavonoids, and 46.75 mg of flavonols	MDA, catalase, glutathione peroxidase, UA, bilirubin	POMj supplementation attenuated oxidative stress (UA) and enhanced antioxidant enzyme activity after intensive weightlifting training session.
Urbaniak et al. (2018) [70]	Double-blind, placebo-controlled	19M well-trained rowers	POMj	2 months, 50 mL daily, total polyphenol content equal to 220 mg/100 g	Antioxidant parameters (TAC), iron metabolism markers (transferrin receptors, iron), inflammation marker (IL-6)	POMj supplementation increased plasma antioxidant potential (TAC) but had no significant effect on iron metabolism markers. Post-exercise IL-6 concentrations increased in both groups but were attenuated by POMj supplementation.
Ammar et al. (2020) [71]	Double-blind, placebo-controlled	9M elite weightlifters	POMj (polyphenol-rich)	250 mL (2.56 g of TPC, 1.08 g of o-DPO), 3 times a day, and 500 mL before the weightlifting session	Hcy, steroidal hormones (testosterone, cortisol), testosterone/cortisol ratio	POMj supplementation reduced post-exercise testosterone and attenuated the increase in Hcy during the 48 h recovery period. No effect on testosterone/cortisol ratio.

Legend: M—male, POMj—pomegranate juice, POMx—pomegranate extract, CK—creatine kinase, IL-6—interleukin-6, CRP—C-reactive protein, RPE—rating of perceived exertion, DOMS—delayed onset of muscle soreness, HR—heart rate, SBP—systolic blood pressure, LDH—lactate dehydrogenase, ASAT—aspartate aminotransferase, TPC—total phenolic content, o-DPO—orthodiphenol, MDA—malonaldehyde, UA—uric acid (oxidative stress), TAC—total antioxidant capacity, Hcy—homocysteine.

**Table 4:** Comprehensive overview of studies about tart cherry juice-based supplementation (*n* = 6).

Study	Study Design	Participants’ Characteristics	Juice Type	Dosage	Measured Outcomes	Findings
Bowtell et al. (2011) [72]	Crossover, randomized	10M athletes (rugby, football, and taekwondo)	MCJ	10 days, 30 mL twice a day, total anthocyanin content was 9.117 mg/mL	MVC, CK, PC, hsCRP, total nitrotyrosine, antioxidant capacity	MCJ improved recovery of MVC and reduced oxidative damage, i.e., PC.
Bell et al. (2015) [73]	Randomized, double-blind, crossover	16M trained cyclists	MTCJ	8 days, 30 mL twice a day, 9.2 mg/mL of anthocyanins	MIVC, cycling efficiency, 6 s peak cycling power, DOMS, inflammation markers (IL-1β, IL-6, TNF-α, hsCRP), oxidative stress (LOOH), muscle damage (CK)	MTCJ supplementation prevented decline in MIVC, improved cycling efficiency, and attenuated IL-6 and hsCRP responses to high-intensity cycling. No significant effect on LOOH or CK.
Bell et al. (2016) [74]	Randomized, double-blind, crossover	16M semi-professional soccer players	MTCJ	8 days, 30 mL twice a day, 9.2 mg/mL of anthocyanins	MIVC, 20 m sprint, CMJ, agility, DOMS, inflammation markers (IL-1-β, IL-6, IL-8, TNF-α, hsCRP), muscle damage (CK), oxidative stress (LOOH)	MTCJ supplementation accelerated recovery of MIVC, CMJ, and agility and reduced DOMS. Acute IL-6 response was attenuated, but no significant effect on CK or LOOH levels.
McCormick et al. (2016) [75]	Randomized, double-blind, crossover	9M water polo athletes	MTCJ	90 mL daily concentrate diluted with water (30 mL serving, i.e., 200 mL beverage), 9.117 mg/mL of anthocyanins	IL-6, CRP, UA, F2-IsoP, DOMS, performance	No significant differences in blood markers, performance, or recovery measures between MTCJ and placebo.
Abbott et al. (2020) [76]	Double-blind, placebo-controlled, crossover	10M professional soccer players	TCJ	2 × 30 mL before and after a 90 min match and 12 and 36 h post-match	CMJ height, RI, DOMS, subjective well-being	TCJ supplementation did not significantly affect muscle function, RI, muscle soreness, or well-being after a soccer match. These findings cast doubt on the efficacy of TCJ as a recovery aid in professional soccer players.
Gao et al. (2024) [77]	Randomized, crossover, counterbalanced, placebo-controlled	12 cyclists (8M + 4F)	TCJ	300 mL/day, twice a day, 4 days before and 2 days after exercise (~9.2 mg/mL of anthocyanins)	MVC, low-frequency fatigue, cycling, performance, substrate metabolism	No significant differences between TCJ and sports drink in time trial performance, muscle soreness, or substrate metabolism.

Legend: M—male, F—female, TCJ—tart cherry juice, MCJ—Montmorency cherry juice, MTCJ—Montmorency tart cherry juice, MVC—maximal voluntary contraction, CK—creatine kinase, PC—protein carbonyls, hsCRP—high-sensitivity C-reactive protein, MIVC—maximal isometric voluntary contractions, DOMS—delayed onset of muscle soreness, IL-6—interleukin-6, TNF—tumor necrosis factor, LOOH—lipid hydroperoxides, CMJ—countermovement jump, CRP—C-reactive protein, UA—uric acid (oxidative stress), F2-IsoP—F2-isoprostane, RI—reactive strength index.

**Table 5:** Comprehensive overview of studies about pickle juice-based supplementation (*n* = 3).

Study	Study Design	Participants’ Characteristics	Juice Type	Dosage	Measured Outcomes	Findings
Miller et al. (2014) [78]	Crossover study	9 physically active individuals (7M + 2F)	PJ	1 mL per kg of body mass, ~0.09 g of acetic acid	Plasma Na^+^, K^+^ concentrations, plasma osmolality, plasma volume changes	Ingesting PJ did not significantly alter plasma Na^+^, K^+^, or osmolality levels. Plasma volume changes were negligible. The study concludes that consuming small amounts of PJ does not fully replenish electrolyte losses after exercise.
Peikert et al. (2014) [79]	Crossover study	9M physically active and euhydrated	PJ	2 mL per kg of body mass, Na^+^: 395 mmol/L, K^+^: 29.5 mmol/L	TTE, core temperature, plasma volume, sweat volume	No significant differences were observed in TTE, core temperature, or sweat volume. Small amounts of PJ do not significantly impact aerobic performance or thermoregulation.
McKenney et al. (2015) [80]	Crossover study	9M euhydrated, physically active	PJ	1 or 2 boluses (1 mL/kg), Na^+^: 530 ± 14 mmol/L, K^+^: 28.8 mmol/L	Plasma Na^+^ and K^+^, plasma osmolality, plasma volume changes	Ingesting up to 2 boluses of PJ during and after exercise caused negligible changes in plasma Na^+^, K^+^, osmolality, and volume. No hyperkalemia occurred.

Legend: M—male, F—female, PJ—pickle juice, Na—sodium, K—potassium, TTE—time to exhaustion.

**Table 6:** Comprehensive overview of studies about watermelon juice-based supplementation (*n* = 6).

Study	Study Design	Participants’ Characteristics	Juice Type	Dosage	Measured Outcomes	Findings
Tarazona-Díaz et al. (2013) [81]	In vivo and in vitro study	7M recreational athletes	WJ (natural and enriched)	500 mL (6 g/500 mL = 1.17 g citrulline from watermelon + 4.83 g of citrulline added)	Muscle soreness, recovery HR	Both natural and enriched WJ reduced muscle soreness and recovery HR 24 h post-exercise. The study concludes that WJ may be a potential functional drink for post-exercise recovery.
Cutrufello et al. (2014) [82]	Randomized, double-blind study	22 participants (11M + 11F)	WJ	710 mL, ~1 g of citrulline or 6 g of L-citrulline, SD	Repetitions completed, time to exhaustion, VO_2_max, anaerobic threshold	No significant effects of L-citrulline or WJ on anaerobic or aerobic exercise performance. The study concludes that a single dose of WJ or L-citrulline is not effective in enhancing performance.
Bailey et al. (2016) [83]	Crossover study	8M healthy recreationally active adults	WJ	16 days, 300 mL/day (11.4 g of L-citrulline and 1.39 g of L-arginine per L)	Plasma NO, BP, muscle oxygenation, TTE	WJ increased plasma NO levels and muscle oxygenation during moderate-intensity exercise but did not improve TTE during severe-intensity exercise. The study concludes that WJ may not effectively enhance endurance performance.
Martínez-Sánchez et al. (2017) [84]	Randomized, double-blind, crossover design	21M amateur runners	WJ (enriched in L-citrulline)	500 mL, 3.45 g of L-citrulline	Muscle soreness, plasma lactate, glucose, lactate dehydrogenase, L-arginine, jump height, HR, perceived exertion	Muscle soreness significantly lower post-race. Plasma lactate concentrations were lower and lactate dehydrogenase and L-arginine concentrations were higher immediately after the race. Jump heights were maintained after WJ.
Gonzalez et al. (2022) [85]	Crossover design	15M resistance-trained	WJ	7 days, 104 mL of WJ concentrate mixed with 355 mL of water, twice a day (2.2 g of L-citrulline)	IMTP test and acute bench press protocol, vessel diameter, muscle oxygenation, and subjective perception	Short-term WJ supplementation resulted in a modest increase in skeletal muscle oxygenation (+4.1%) compared to placebo. However, it did not lead to significant improvements in isometric strength, bench press performance, vascular diameter, or overall muscle oxygenation in resistance-trained men.
Aghabeighiamin and Azizi (2025) [86]	RCT	25F elite taekwondo athletes	WJ	6 weeks, 500 mL daily, lycopene: 3.38–11.34 mg	TAC	WJ supplementation significantly increased TAC after 6 weeks. The findings suggest that WJ may be beneficial in reducing oxidative stress in elite athletes.

Legend: M—male, F—female, WJ—watermelon juice, HR—heart rate, SD—single-dose, VO_2_max—maximal oxygen uptake, NO—nitric oxide, BP—blood pressure, TTE—time to exhaustion, IMTP—isometric mid-thigh pull, RCT—randomized controlled trial, TAC—total antioxidant capacity.

**Table 7:** Comparative effects of beetroot, pomegranate, cherry, watermelon, and pickle juices on key physiological outcomes in athletes.

Juice Type	Inflammation	Oxidative Stress	DOMS	Performance	Key Notes
BRJ	Mixed (↑ NO may help, but inconsistent) [37,39,45]	Limited evidence [42]	Modest relief in some trials [50]	Strong for endurance, weaker for sprinting/elite [38,45,46]	Dose-dependent; best when preloaded 2–3 h before
POMj	↓ IL-6, ↓ CRP, ↓ Hcy [68,69,71]	↓ MDA, ↑ catalase, ↑ TAC [69,70]	Effective in upper-body eccentric soreness [66,67]	Mixed; mainly recovery-focused [68,71]	Strongest for oxidative and endocrine modulation
CJ	↓ IL-6, CRP in many studies [72,73,74]	↓ Lipid peroxidation [72]	↓ Soreness, especially preloading [73,74]	Inconsistent across sports [76]	Best when consumed on days before and after exercise
PJ	No measurable effect [78,80]	Not assessed	Not directly measured	No significant impact [78,79]	Anecdotal relief of cramps not yet confirmed
WJ	Limited evidence [86]	↑ TAC, ↓ lactate (chronic use) [83,86]	↓ Soreness with enriched formulations [81]	Inconsistent, no VO_2_max changes [82,85]	Pasteurization reduces citrulline bioavailability

Legend: BRJ—beetroot juice, POMj—pomegranate juice, CJ—cherry juice, WJ—watermelon juice, PJ—pickle juice, DOMS—delayed onset of muscle soreness, NO—nitric oxide, IL-6—interleukin-6, CRP—C-reactive protein, Hcy—homocysteine, MDA—malonaldehyde, TAC—total antioxidant capacity, VO_2_max—maximal oxygen uptake, ↑—increase, ↓—reduction.

## Supplementary Materials

The following supporting information can be downloaded at: https://www.mdpi.com/article/10.3390/sports13080269/s1. Supplementary File S1. PRISMA 2020 checklist, Supplementary File S2. INPLASY protocol, Supplementary Table S1. PEDro scale evaluation.

## Abbreviations

The following abbreviations are used in this manuscript:

NO	Nitric Oxide
BRJ	Beetroot Juice
POMj	Pomegranate Juice
IL-6	Interleukin-6
TNF	Tumor Necrosis Factor
TCJ	Tart Cherry Juice
PJ	Pickle Juice
WJ	Watermelon Juice
RCTs	Randomized Controlled Trials
INPLASY	International Platform of Registered Systematic Review and Meta-Analysis Protocols
PEDro	Physiotherapy Evidence Database Scale
M	Male
F	Female
NO_2_^−^	Nitrite
NO_3_^−^	Nitrate
VO_2_	Oxygen Uptake
TT	Time Trial
YYIR1	Yo-Yo Intermittent Recovery level 1
SD	Single-Dose
DD	Double-Dose
AEC	Aerobic Energy Cost
VCO_2_	Carbon Dioxide production
VE	Minute Ventilation
MVC	Maximal Voluntary Contraction
MIVC	Maximal Isometric Voluntary Contraction
SJ	Squat Jump
CMJ	Countermovement Jump
RI	Reactive Strength Index
PPT	Pressure Pain Threshold
CK	Creatine Kinase
CRP	C-Reactive Protein
hsCRP	High-Sensitivity C-Reactive Protein
PC	Protein Carbonyls
LOOH	Lipid Hydroperoxides
SaO_2_	Oxygen Saturation
HR	Heart Rate
DEXA	Dual X-Ray Absorptiometry
RPE	Rating of Perceived Exertion
HI	High
LO	Low
KNO_3_	Potassium Nitrate
SIT	Sprint Interval Training
BP	Blood Pressure
SBP	Systolic Blood Pressure
GAE	Gallic Acid Equivalent
LDH	Lactate Dehydrogenase
DOMS	Delayed-Onset Muscle Soreness
TTE	Time to Exhaustion
XC	Cross-Country
RTF	Repetitions to Failure
TSI	Tissue Saturation Index
VAS	Visual Analog Scale
TQR	Total Quality Recovery
VO_2_max	Maximal Oxygen uptake
MBT	Medicine Ball Throw
VJH	Vertical Jump Height
BF%	Body Fat Percentage
POMx	Pomegranate Extract
ASAT	Aspartate Aminotransferase
TPC	Total Phenolic Content
o-DPO	Orthodiphenol
MDA	Malonaldehyde
UA	Uric Acid
TAC	Total Antioxidant Capacity
Hcy	Homocysteine
MCJ	Montmorency Cherry Juice
MTCJ	Montmorency Tart Cherry Juice
F2-IsoP	F2-Isoprostane
Na	Sodium
K	Potassium
IMTP	Isometric Mid-Thigh Pull
BMX	Bicycle Motocross
GPS	Global Positioning System
