# Supplementary material for: Juice-Based Supplementation Strategies for Athletic Performance and Recovery: A Systematic Review
Source: Sports (Basel). 2025 Aug 14;13(8):269. doi: 10.3390/sports13080269 (PMC12389966; doi:10.3390/sports13080269)
Supplement: Supplementary file 1 [file sports-13-00269-s001.zip › Supplementary File S2-INPLASY protocol.pdf]

International Platform of Registered Systematic Review and Meta-analysis Protocols

INPLASY

Juice-Based Supplementation Strategies For Athletic Performance And Recovery: A Systematic Review

INPLASY202560053
doi: 10.37766/inplasy2025.6.0053
Received: 12 June 2025
Published: 12 June 2025

Vitošević, B; Filipović, M; Popović, L; Sterkowicz-Przybycień, K; Purenović-Ivanović, T.

Corresponding author:
Milica Filipović

milica.bojovic@pr.ac.rs

Author Affiliation:
Faculty of sport and phyisical education, University of Pristina - Kosovska Mitrovica.

Vitošević et al. INPLASY protocol 202560053. doi:10.37766/inplasy2025.6.0053 Downloaded from https://inplasy.com/inplasy-2025-6-0053/

ADMINISTRATIVE INFORMATION

Support - No specific financial support has been received for this review.

Review Stage at time of this submission - Completed but not published.

Conflicts of interest - None declared.

INPLASY registration number: INPLASY202560053

Amendments - This protocol was registered with the International Platform of Registered Systematic Review and Meta-Analysis Protocols (INPLASY) on 12 June 2025 and was last updated on 12 June 2025.

INTRODUCTION

Review question / Objective What is the effectiveness of different types of natural juices (beetroot, pomegranate, cherry, watermelon, and pickle juice) on athletic performance and recovery in athletes?

Rationale Natural juices rich in antioxidants and bioactive compounds are increasingly used in sports nutrition due to their potential ergogenic and recovery-enhancing effects. Given the inconsistent findings in the literature, a systematic synthesis is needed to evaluate their true impact on performance and recovery.

Condition being studied Muscle fatigue, delayed onset muscle soreness (DOMS), oxidative stress, and exercise-induced performance decline.

METHODS

Search strategy
Databases: PubMed, Scopus, Web of Science.
Keywords: “juice”, “beetroot”, “pomegranate”, “cherry”, “watermelon”, “pickle juice”, “exercise”, “recovery”, “performance”.
Boolean operators used (AND, OR).
Date range: 2010–2025.
Language: English only.

Participant or population Healthy adult athletes (male and female), aged 18–45 years, including both recreational and elite level.

Intervention Dietary supplementation with natural juices: beetroot, cherry, pomegranate, watermelon, and pickle juice.

Comparator Placebo beverages, water, or no intervention.

Study designs to be included Randomized controlled trials (RCTs), crossover studies.

Eligibility criteria This systematic review included studies that met the following inclusion criteria: a)studies involving human participants (athletes or physically active individuals); b) studies using supplementation of the specified juices; c) randomized controlled trials (RCTs), cohort studies, cross-sectional studies, and case studies; d) studies reporting outcomes related to exercise performance, muscle soreness, inflammation or recovery; e) studies published in English. Exclusion criteria were: a) animal studies; b) meta-analyses, systematic reviews, and other types of reviews; c) studies involving synthetic supplements other than liquid (capsule, powder) or multi-ingredient products not isolated to the specified juices; d) studies without data on performance or recovery outcomes. Data were extracted using a standardized data extraction form, which included the following: Authors (year, country), Study design, Participant characteristics (age, gender, training status), Juice type, dosage, duration, and frequency, Outcomes measured (muscle soreness, inflammation, performance metrics) and Findings.
The methodological quality of the included studies was assessed using the Physiotherapy Evidence Database (PEDro) scale, which is a valid tool for quality assessment in clinical studies . Due to the high heterogeneity in study designs, populations, interventions, and outcomes, a meta-analysis was not performed. Instead, a qualitative synthesis was used to interpret and compare findings across the included studies.

Information sources A comprehensive literature search was conducted at the beginning of May 2025 using the following databases: PubMed, Scopus, Web of Science. The search incorporated a combination of keywords and Boolean operators (AND/OR), focusing on: (Supplements OR Juices) AND (Athletes OR Recovery OR Exercise Performance), (Beetroot OR Pomegranate OR Cherry OR Pickle OR Watermelon), (Antioxidants OR Inflammation OR Muscle Soreness OR Cramp).

Main outcome(s) Performance: VO_2max, power, endurance, sprint, explosiveness. Recovery: DOMS, biomarkers of inflammation and oxidative stress.

Additional outcome(s) Ratings of perceived exertion (RPE), subjective recovery scores, gastrointestinal effects.

Data management Data will be stored in Excel spreadsheets. Two reviewers will independently input and verify data.

Quality assessment / Risk of bias analysis PEDro scale will be used. Two reviewers will assess independently; a third will resolve disagreements.

Strategy of data synthesis The study selection process was documented using the PRISMA flowchart, detailing the number of studies identified, screened, excluded, and included in the review. Data were extracted using a standardized data extraction form, which included the following: Authors (year, country), Study design, Participant characteristics (age, gender, training status), Juice type, dosage, duration, and frequency, Outcomes measured (muscle soreness, inflammation, performance metrics) and Findings. The methodological quality of the included studies was assessed using the Physiotherapy Evidence Database (PEDro) scale, which is a valid tool for quality assessment in clinical studies. All authors independently assessed the quality of the selected studies, and consensus was reached by mutual agreement. Due to the high heterogeneity in study designs, populations, interventions, and outcomes, a meta-analysis was not performed. Instead, a qualitative synthesis was used to interpret and compare findings across the included studies.

Subgroup analysis Although no formal statistical subgroup analysis was performed, a qualitative synthesis allowed for comparative evaluation based on juice type (beetroot, pomegranate, cherry, watermelon, pickle), participant characteristics (recreational vs. elite athletes), and duration of supplementation (acute vs. chronic). This approach enabled identification of outcome patterns depending on supplementation context.

Sensitivity analysis Due to the qualitative nature of the synthesis and the high heterogeneity among included studies, a formal sensitivity analysis was not conducted. Instead, consistency of findings was examined by comparing results across studies of varying methodological quality (based on the PEDro scale), as well as differing designs and populations.

Language restriction English only.

Country(ies) involved Serbia, Poland.

Keywords juice; athletes; nutrition; exercise; ergogenic aid.

Vitošević et al. INPLASY protocol 202560053. doi:10.37766/inplasy2025.6.0053 Downloaded from https://inplasy.com/inplasy-2025-6-0053/

Contributions of each author

Author 1 - Biljana Vitošević - Conceived the review, performed the literature search, and drafted the manuscript.
Email: biljana.vitosevic@pr.ac.rs
Author 2 - Milica Filipović - Screened studies, extracted data, and contributed to quality assessment.
Email: milica.bojovic@pr.ac.rs
Author 3 - Ljiljana Popović - Assisted in data interpretation and participated in quality scoring(PEDro).
Email: ljiljana.popovic@med.pr.ac.rs
Author 4 - Katarzyna Sterkowicz-Przybycień - Validated methods and contributed to manuscript revisions.
Email: katarzyna.sterkowicz@awf.krakow.pl
Author 5 - Tijana Purenović - Ivanović - Supervised the project, coordinated contributions, and is the corresponding author.
Email: tijanapurenovic@gmail.com

Vitošević et al. INPLASY protocol 202560053. doi:10.37766/inplasy2025.6.0053 Downloaded from https://inplasy.com/inplasy-2025-6-0053/
