# Supplementary material for: Juice-Based Supplementation Strategies for Athletic Performance and Recovery: A Systematic Review
Source: Sports (Basel). 2025 Aug 14;13(8):269. doi: 10.3390/sports13080269 (PMC12389966; doi:10.3390/sports13080269)
Supplement: Supplementary file 1 [file sports-13-00269-s001.zip › Supplementary Table S1-PEDro scale evaluation.pdf]

Supplementary Table 1. PEDro Scale Evaluation – Quality assessment

Included studies	Criteria												Study quality
	1	2	3	4	5	6	7	8	9	10	11	Total	
Christensen et al. [37]	Yes	1	0	1	1	0	1	1	0	1	1	7	good
Wylie et al. [38]	Yes	1	0	1	1	0	1	1	1	1	1	7	good
Martin et al. [39]	Yes	1	0	1	1	0	1	1	0	1	1	7	good
Pinna et al. [40]	Yes	1	0	1	1	0	1	1	1	1	1	8	good
Thompson et al. [41]	Yes	1	0	1	1	0	1	1	1	1	1	8	good
Clifford et al. [42]	Yes	1	0	1	0	0	1	1	1	1	1	7	good
Patrician & Schagatay [43]	Yes	1	0	1	1	0	1	1	1	1	1	8	good
Thompson et al. [44]	Yes	1	0	1	1	0	1	1	1	1	1	8	good
Wylie et al. [45]	Yes	1	0	1	1	0	1	1	1	1	1	8	good
Jonvik et al. [46]	Yes	1	1	1	1	0	1	1	1	1	1	9	excellent
Richard et al. [47]	Yes	1	1	1	1	0	1	1	1	1	1	9	excellent
Thompson et al. [48]	Yes	1	1	1	1	0	1	1	1	1	1	9	excellent
Esen et al. [49]	Yes	1	0	1	0	0	1	1	0	1	1	6	good
Daab et al. [50]	Yes	1	0	1	1	0	1	1	1	1	1	8	good
Fernández-Elías et al. [51]	Yes	1	0	1	0	0	1	1	1	1	1	7	good
Garnacho-Castaño et al. [52]	Yes	1	0	1	0	0	1	1	1	1	1	7	good
Esen et al. [53]	Yes	1	0	1	0	0	1	1	1	1	1	7	good
Esen et al. [54]	Yes	1	1	1	1	1	1	0	1	1	1	9	excellent
Giv et al. [55]	Yes	1	0	1	0	0	1	1	1	1	1	7	good
Huang et al. [56]	Yes	1	0	1	0	0	1	1	1	1	1	7	good
Jurado-Castro et al. [57]	Yes	1	1	1	1	0	1	1	1	1	1	9	excellent
Tan et al. [58]	Yes	1	0	1	1	0	1	1	0	1	1	7	good
Esen et al. [59]	Yes	1	0	1	1	0	1	1	0	1	1	7	good
Hemmatinafar et al. [60]	Yes	1	0	1	1	0	1	1	1	1	1	8	good
Moreno-Heredero et al. [61]	Yes	1	0	1	1	0	1	1	1	1	1	8	good
Neteca et al. [62]	Yes	1	0	1	1	0	1	1	1	1	1	8	good
Tan et al. [63]	Yes	1	0	1	1	0	1	1	1	1	1	8	good
Zhang et al. [64]	Yes	1	0	1	1	0	1	1	1	1	1	8	good
Liu et al. [65]	Yes	1	0	1	1	0	1	1	1	1	1	8	good
Trombold et al. [66]	Yes	0	0	1	1	0	1	1	0	1	1	6	good
Trombold et al. [67]	Yes	0	0	1	1	0	1	1	0	1	1	6	good
Ammar et al. [68]	Yes	1	1	1	0	0	1	1	1	1	1	8	good
Ammar et al. [69]	Yes	1	0	1	0	0	1	1	0	1	1	6	good
Urbaniak et al. [70]	Yes	0	0	1	1	0	1	1	0	1	1	6	good
Ammar et al. [71]	Yes	1	0	1	0	0	1	1	1	1	1	7	good
Bowtell et al. [72]	Yes	1	1	1	1	0	1	1	1	1	1	9	excellent
Bell et al. [73]	Yes	1	0	1	1	0	0	1	1	1	1	7	good
Bell et al. [74]	Yes	1	1	1	1	0	1	1	1	1	1	9	excellent
McCormick et al. [75]	Yes	1	0	1	1	0	1	1	1	1	1	8	good
Abbott et al. [76]	Yes	1	0	1	0	0	1	1	1	1	1	7	good
Gao et al. [77]	Yes	1	0	1	1	0	1	1	1	1	1	8	good
Miller et al. [78]	Yes	1	1	1	1	0	1	1	1	1	1	9	excellent

Peikert et al. [79]	Yes	1	0	1	1	0	1	1	1	1	1	8	good
McKenney et al. [80]	Yes	1	0	1	0	0	1	1	1	1	1	7	good
Tarazona-DÍaz et al. [81]	Yes	1	1	1	1	0	1	1	1	1	1	9	excellent
Cutrufello et al. [82]	Yes	1	1	1	1	0	1	1	1	1	1	9	excellent
Bailey et al. [83]	Yes	1	0	1	1	0	1	1	1	1	1	8	good
Martínez-Sánchez et al. [84]	Yes	1	0	1	1	0	1	1	1	1	1	8	good
Gonzalez et al. [85]	Yes	1	1	1	1	0	1	1	1	1	1	9	excellent
Aghabeighiamin & Azizi [86]	Yes	1	1	1	1	0	1	1	1	1	1	9	excellent
**TOTAL**												**7.7/10**	

Scoring: 'yes' = 1, 'no' = 0, 'unable to determine' = 0; Criteria: 1) Eligibility criteria specified; 2) Random allocation; 3) Concealed allocation; 4) Baseline comparability; 5) Blinding of subjects; 6) Blinding of therapists; 7) Blinding of assessors; 8) Measures of at least one key outcome obtained from more than 85% of subjects initially allocated to groups; 9) Intention-to-treat analysis; 10) Between-group statistical comparisons reported for at least one key outcome; 11) Point measures and measures of variability for at least one key outcome. Total Score: /10
